# Comprehensive Review on Doping Strategies for Two-Dimensional Tungsten Diselenide

**DOI:** 10.3390/ijms27156552

**Published:** 2026-07-23

**Authors:** Donghun Lee

**Affiliations:** Department of Chemistry, Kookmin University, Seoul 02707, Republic of Korea; donghunlee@kookmin.ac.kr

**Keywords:** tungsten diselenide, two-dimensional materials, ambipolar transport, doping

## Abstract

Two-dimensional transition metal dichalcogenides are being studied as channel materials for beyond-silicon electronics because their atomic-scale thickness enables strong electrostatic control. Among these materials, tungsten diselenide (WSe_2_) is particularly attractive because it exhibits ambipolar transport. Despite this advantage, practical WSe_2_ transistors remain constrained by Fermi-level pinning at metal contacts, contact-dominated carrier injection, and defect-induced variability, making controlled doping a central issue. This review examines doping strategies developed for WSe_2_, focusing on methods compatible with atomically thin van der Waals semiconductors. The discussion covers surface charge-transfer doping by molecular adsorbates, Lewis acids, and alkali metals, as well as defect-mediated chemisorption, self-limiting oxide interfacial layers, and in situ vacancy engineering during growth. Particular attention is given to the thermodynamic mechanisms of charge transfer, the distinction between contact-selective and channel-selective doping, and the trade-offs between degenerate and non-degenerate doping. The effects of doping are also discussed, including contact-resistance reduction through Schottky-barrier narrowing, threshold-voltage control, apparent mobility enhancement through impurity screening and defect passivation, and thermal and temporal stability. The review further summarizes how controlled doping has enabled complementary metal-oxide-semiconductor logic circuits and identifies the remaining challenges in air stability, selective-area patterning, scalable synthesis, and low-temperature integration for monolithic three-dimensional electronic devices.

## 1. Introduction

### 1.1. Evolution of 2D Transition Metal Dichalcogenides (TMDs) in Next-Generation Electronics

**Figure 1:**
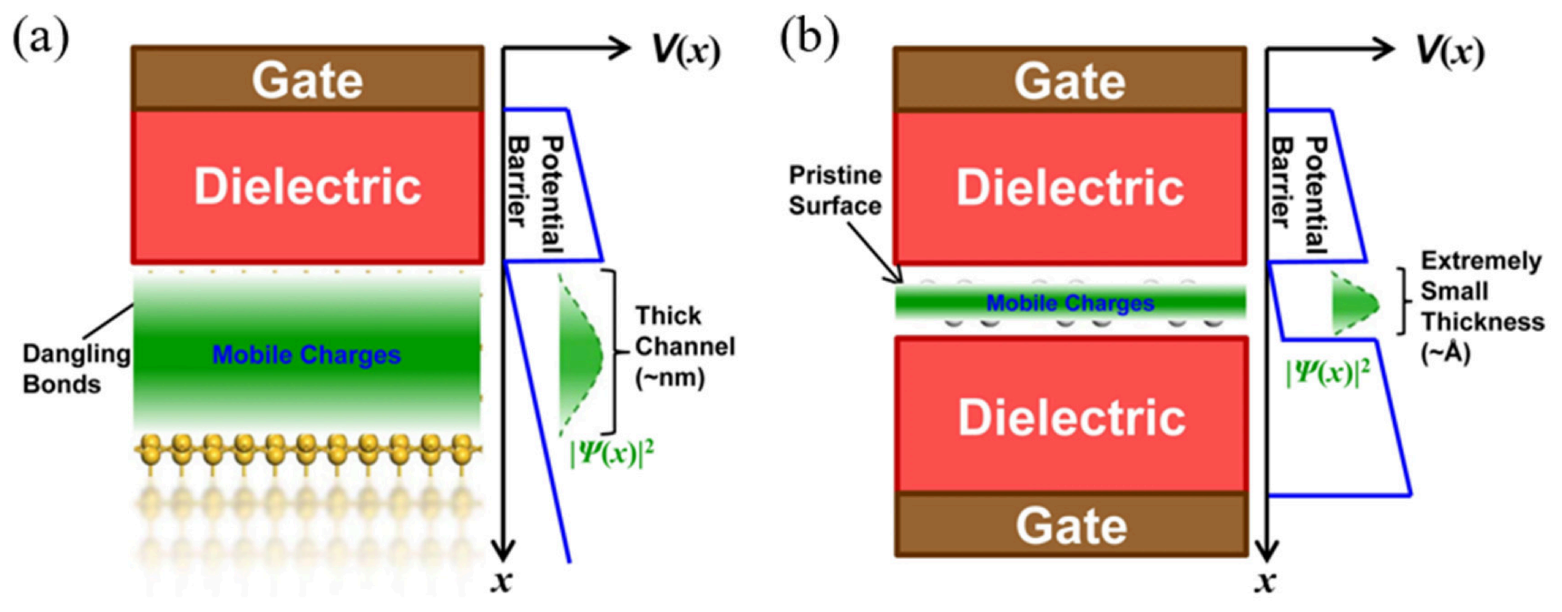
Schematic comparison of conventional bulk and atomically thin semiconductors: (**a**,**b**) Spatial distributions of mobile carriers in (**a**) 3D and (**b**) 2D semiconductor channels under gate bias. *V*(*x*) denotes the gate-induced electrostatic potential along the out-of-plane direction, whereas |*Ψ*(*x*)|^2^ represents the corresponding carrier probability density. Reproduced with permission [7].

The continuous miniaturization of Si-based complementary metal-oxide-semiconductor (CMOS) architecture has historically driven the exponential growth of modern computing [1,2,3]. However, as the semiconductor industry enters sub-nanometer technology nodes, Si faces fundamental scaling limitations that are increasingly difficult to overcome. At these extreme dimensions, conventional three-dimensional (3D) bulk channels suffer from severe short-channel effects, surface-roughness scattering, and excessive power consumption [4,5]. As a result, traditional Moore’s law scaling is approaching its physical limits. To sustain device scaling and performance improvement, the scientific community has increasingly focused on two-dimensional (2D) TMDs [6]. Their dangling-bond-free basal planes enable atomically abrupt van der Waals interfaces, allowing diverse heterostructures and functional devices. In addition, 2D semiconductors provide strong gate control over carrier transport because the channel thickness is confined to one or a few atomic layers (Figure 1) [7,8,9]. These structural characteristics suppress short-channel effects and establish a robust material platform for next-generation, ultra-high-density electronic devices.

Beyond traditional logic channels, 2D TMDs are emerging as promising candidates for next-generation in-memory computing [10]. For instance, various 2D-material-based memories using vacancy configurations, ion migration, or spin states have been developed to implement brain-inspired computing hardware. Furthermore, controlling stacking sequences in layered ferroelectrics, such as α-In_2_Se_3_, enables robust polarization switching [11]. To fully realize the potential of 2D materials, methods to synthesize wafer-scale films must be developed. Furthermore, polycrystalline films inherently suffer from critical issues associated with high-density grain boundaries, which significantly degrade charge transport and increase device-to-device performance variability. To address these limitations, Wang et al. demonstrated the direct epitaxial growth of two-inch single-crystal WS_2_ monolayers on vicinal *a*-plane sapphire substrates [12]. In this growth process, the interaction with the sapphire surface guides WS_2_ domains into two antiparallel orientations, while the atomic step edges break this symmetry to favor single-orientation growth. This unidirectional alignment enables the seamless stitching of adjacent islands, thereby forming a continuous single-crystal film without grain boundaries. Furthermore, beyond conventional electronics, 2D semiconductors have emerged as an important material platform for exploring quantum transport phenomena. A representative example is the implementation of a charge-transfer contact structure in a monolayer WSe_2_, which achieves high-concentration hole doping localized to the contact region while maintaining an intrinsic channel [13]. This strategy drastically reduces contact resistance and achieves a remarkably high hole mobility of 80,000 cm^2^ V^−1^ s^−1^ at low temperatures. This breakthrough enables electrical transport measurements of strongly correlated electronic states and quantum phases, including the low-temperature metal-insulator transition at low carrier densities and the fractional quantum Hall effect.

### 1.2. Unique Properties of Tungsten Diselenide (WSe_2_)

**Figure 2:**
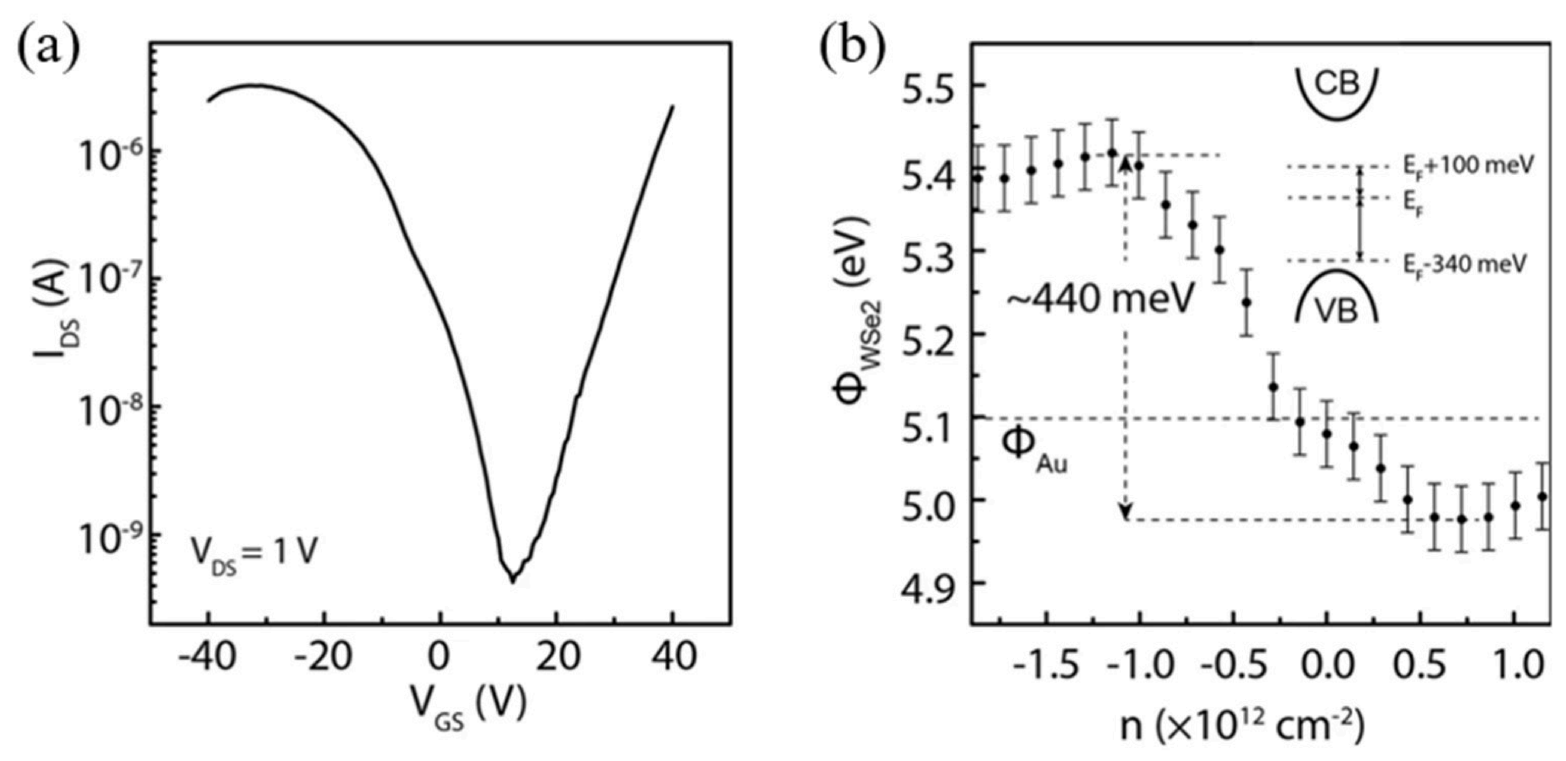
(**a**) Representative transfer curve of an ambipolar WSe_2_ field-effect transistor, showing gate-controlled electron- and hole-transport regimes. (**b**) Carrier-density-dependent variation in the WSe_2_ work function. The inset illustrates the corresponding E_F_ displacement. Reproduced with permission [17].

Among semiconducting group-VI TMDs, molybdenum disulfide (MoS_2_) first attracted broad interest in 2D electronics. However, MoS_2_ transistors often favor electron conduction because defect-related states and contact-induced pinning tend to place the Fermi level (E_F_) close to the conduction band minimum [14]. In contrast, recent contact-engineering studies further indicate that the E_F_ of WSe_2_ is pinned near the mid-gap, unlike in MoS_2_ or WS_2_ (Figure 2). Therefore, single- and few-layer WSe_2_ devices commonly show ambipolar transport behavior [15,16,17]. From a device-engineering perspective, such symmetric charge transport is a key advantage. It enables selective control of either conduction pathway via contact design, electrostatic gating, or chemical doping on a single material platform. In addition, bulk WSe_2_ is an indirect-gap semiconductor with a gap near 1.2 eV, whereas monolayer WSe_2_ exhibits a direct optical transition around 1.65 eV [18]. Spin- and angle-resolved photoemission measurements have also resolved a valence-band spin splitting of roughly 0.5 eV, which is several times larger than the approximately 0.15 eV commonly discussed for monolayer MoS_2_ [19,20]. This large splitting originates from the strong spin–orbit interaction associated with W-derived d orbitals and the broken inversion symmetry of the monolayer crystal. The coexistence of ambipolar transport and a thickness-dependent bandgap makes WSe_2_ a versatile platform. Consequently, it has emerged as a key candidate for monolithic CMOS logic as well as advanced research in valleytronics, spintronics, and optoelectronics.

### 1.3. The Critical Need for Controllable Doping in WSe_2_

Controllable doping in WSe_2_ is required for two distinct aspects of transistor operation: carrier injection at the source/drain contacts and electrostatic modulation of the channel. In practice, most reported WSe_2_ doping methods do not involve classical substitutional doping of the completed lattice; instead, they rely on surface charge transfer, chemisorption, oxide-mediated charge transfer, or defect engineering. When pristine WSe_2_ is contacted with metal electrodes, the dependence of the Schottky barrier on the metal work function is weakened by interfacial conditions, metal-induced gap states (MIGS), and defect-induced gap states (DIGS). As a result, the barrier height often deviates from the Schottky–Mott limit [21,22]. Deposited metal/WSe_2_ contacts, therefore, often limit carrier injection and can even produce unintended carrier polarity. For this reason, local doping near the contacts is one of the most practical routes to narrow the depletion region, increase tunneling through the barrier, and suppress contact-limited transport [23,24].

At the same time, doping plays an equally critical role in modulating the intrinsic semiconducting channel. Gate operation is governed by the electrostatic state of the active channel. Pristine or lightly doped WSe_2_ often exhibits ambipolar transport, which is undesirable for reproducible circuit design. By intentionally injecting carriers into the channel region, the channel E_F_ shifts toward the conduction-band edge (E_C_) or valence-band edge (E_V_). This changes the equilibrium carrier density, shifts the threshold voltage (V_th_), and suppresses unwanted ambipolar conduction [25,26]. Such control is particularly important for complementary logic circuits because CMOS requires not only low-resistance contacts but also matched V_th_ values and comparable drive currents or transconductance in p- and n-channel devices. Therefore, the key issue in WSe_2_ doping is not only the carrier concentration but also the spatial distribution of the induced charge. This article provides a comprehensive and critical evaluation of state-of-the-art doping techniques for 2D WSe_2_.

## 2. Fundamentals of Conventional Doping Mechanisms in 2D WSe_2_

### 2.1. Limitations of Conventional 3D Doping Techniques (Ion Implantation) for Atomically Thin Materials

**Figure 3:**
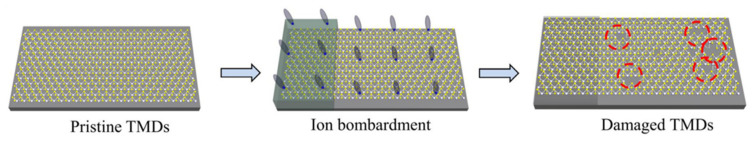
Schematic illustration of the structural limitations of conventional ion-implantation doping in atomically thin semiconductors, showing how energetic ion bombardment induces lattice disorder, defect formation, and structural damage in the 2D lattice. Reproduced with permission [28].

For decades, conventional doping in bulk semiconductors has commonly been implemented by ion implantation, in which energetic dopant ions are introduced into a 3D crystal and subsequently activated by thermal annealing. However, applying this established method to 2D TMDs presents severe physical difficulties [27,28]. Monolayer WSe_2_ is only about 0.7 nm thick. Under the kinetic energies typically required for ion bombardment, the atomically thin lattice lacks sufficient stopping power to confine implanted ions within a single atomic layer. Instead of incorporating dopant atoms controllably, collision cascades generated during implantation can cause irreversible structural damage to the WSe_2_ lattice (Figure 3). Consequently, bombardment-induced defects act as scattering centers and deep-level charge traps rather than simple carrier-density modulators. These defects suppress the intrinsic carrier mobility of WSe_2_ and degrade electrostatic gate control, thereby eliminating many of the inherent transport advantages of 2D semiconductors. These limitations have driven a necessary paradigm shift toward non-destructive modulation strategies, including surface charge transfer by molecular doping, localized native-oxide interfacial layers, and in situ defect engineering.

### 2.2. Mechanistic Overview of Various Doping Strategies

**Figure 4:**
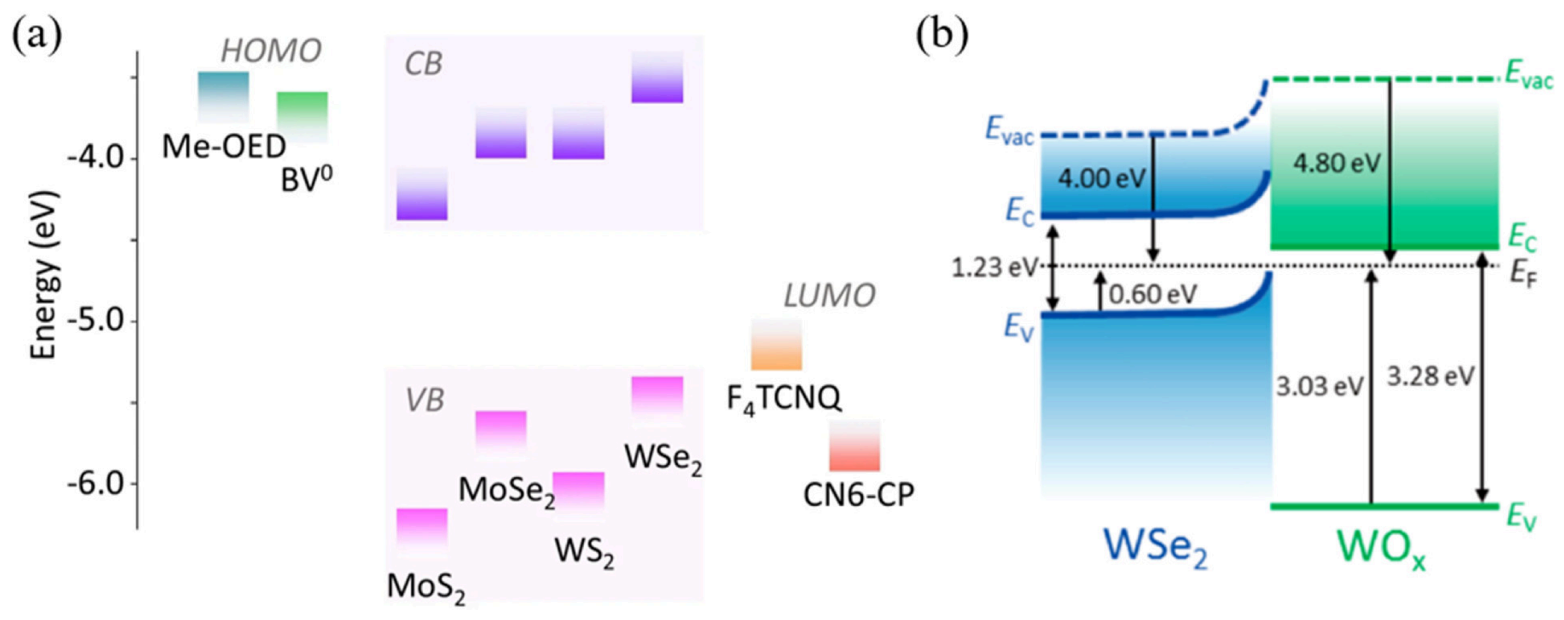
(**a**) Schematic energy-level diagrams showing the relative alignment between the band edges of 2D semiconductors and the LUMO and HOMO levels of selected molecular dopants. CB and VB denote the conduction band and valence band, respectively. Reproduced with permission [29]. (**b**) Energy-band diagram of the WO_x_/WSe_2_ heterointerface. Reproduced with permission [33].

Doping strategies for 2D WSe_2_ predominantly rely on physical and chemical mechanisms of carrier modulation: physisorption, chemisorption, oxide-mediated, and defect engineering. First, physisorption-based surface charge transfer doping (SCTD) serves as a relatively non-destructive approach where organic or inorganic molecules physically adsorb onto the pristine WSe_2_ basal plane. Charge transfer is governed by the energy level alignment of the lowest unoccupied molecular orbital (LUMO) and highest occupied molecular orbital (HOMO) of the dopants relative to the channel band edges, without causing structural damage (Figure 4a) [29,30,31]. Second, chemisorption involves the formation of robust covalent bonds where highly reactive gas molecules selectively bind to selenium vacancies (V_Se_), pinning or shifting the E_F_ near the band edge [16,24]. Third, oxide-mediated doping utilizes controlled, self-limiting oxidation of the top surface layers to form a high-work-function transition-metal suboxide, inducing spontaneous electron extraction from the underlying WSe_2_ (Figure 4b) [32,33]. Finally, defect engineering relies on the intentional generation or healing of atomic vacancies, introducing localized shallow defect levels to modulate the majority-carrier type [34].

## 3. Advanced Doping Strategies for WSe_2_

### 3.1. p-Type Doping Approaches

#### 3.1.1. Organic Acceptor Molecules with High Stability

**Figure 5:**
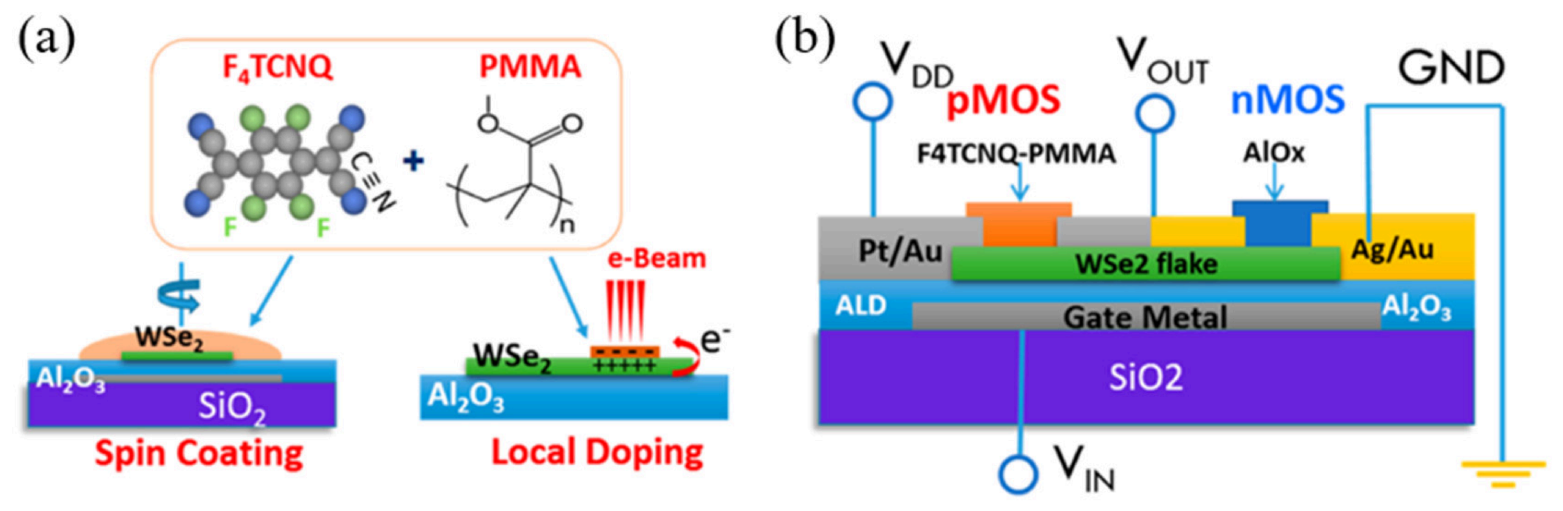
(**a**) Schematic illustration of a localized and environmentally robust p-doping scheme using an F_4_TCNQ-PMMA composite. (**b**) Cross-sectional representation of WSe_2_ CMOS inverter circuitry composed of F_4_TCNQ-PMMA-doped p-channel metal oxide semiconductor field-effect transistors (MOSFETs) and an AlO_x_-encapsulated n-channel MOSFET. Reproduced with permission [25].

Among molecular acceptors examined for p-type modulation of WSe_2_, F_4_TCNQ remains one of the clearest examples of noncovalent charge-transfer doping [25]. The thermodynamic driving force for F_4_TCNQ as a high-efficiency surface charge-transfer dopant originates from its LUMO. Located about 5.2 eV below the vacuum level, this LUMO is energetically lower than the E_V_ of WSe_2_. Consequently, spontaneous interfacial electron transfer occurs from WSe_2_ to adsorbed F_4_TCNQ molecules, leading to high-density hole accumulation in the WSe_2_ channel. Despite its strong doping capability, F_4_TCNQ suffers from volatility at elevated temperatures and can show performance degradation under ambient conditions. To address this instability, F_4_TCNQ molecules have been incorporated into a poly(methyl methacrylate) (PMMA) matrix (Figure 5) [25]. This encapsulation strategy immobilizes dopants on the WSe_2_ surface, maintains p-type characteristics and high on-current for several weeks, and enables precise spatial patterning by electron-beam lithography. Through such area-selective doping, a monolithic CMOS logic inverter was fabricated on a single WSe_2_ flake.

#### 3.1.2. Lewis Acid-Based Doping

Beyond organic electron-withdrawing molecules, Lewis acids provide another non-destructive method for SCTD. Among Lewis acid dopants, transition-metal chlorides such as iron(III) chloride (FeCl_3_) and platinum(IV) chloride (PtCl_4_) serve as strong electron acceptors [35,36,37]. When applied to the WSe_2_ surface, these metal compounds spontaneously withdraw electrons from WSe_2_ because of their high electron affinity. As a result, holes are generated in WSe_2_ without covalently damaging the basal plane. In solution-processed WSe_2_ thin-film transistors, FeCl_3_ treatment lowered E_F_, as measured by UPS, from −4.11 to −4.55 eV and reduced the E_V_−E_F_ separation from 1.47 to 0.98 eV [35]. This treatment increased the hole concentration to 10^12^ cm^−2^. A later study on few-layer WSe_2_ doped with a higher-concentration FeCl_3_ solution reported a hole concentration of 10^13^ cm^−2^ and a sixfold increase in conductivity [36].

**Figure 6:**
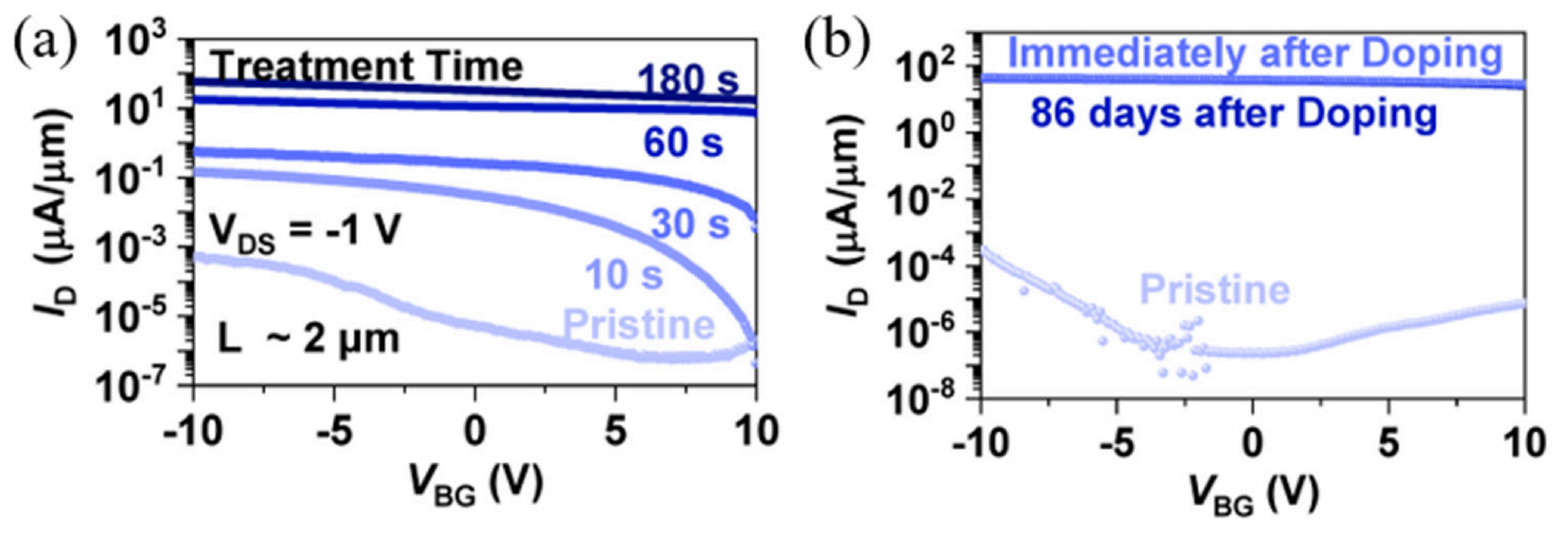
(**a**) Drain current as a function of back-gate bias for WSe_2_ transistors treated with PtCl_4_ for treatment times ranging from 0 to 180 s. (**b**) Transfer characteristics of a PtCl_4_-doped WSe_2_ device measured immediately after PtCl_4_ treatment and after 86 days of doping. Reproduced with permission [37].

PtCl_4_-based doping has been developed as a stable, CMOS-compatible alternative. The underlying mechanism relies on the reduction potential of Pt ions (Pt^2+^ + 2e^−^ → Pt^0^), which is energetically lower than the E_V_ of WSe_2_. PtCl_4_-doped WSe_2_ devices exhibit exceptional environmental durability, maintaining nearly identical transfer characteristics after approximately three months of ambient air exposure (Figure 6) [37]. Furthermore, the doped state remains stable down to 78 K under high-vacuum conditions.

#### 3.1.3. Covalent Functionalization and Chemisorption

**Figure 7:**
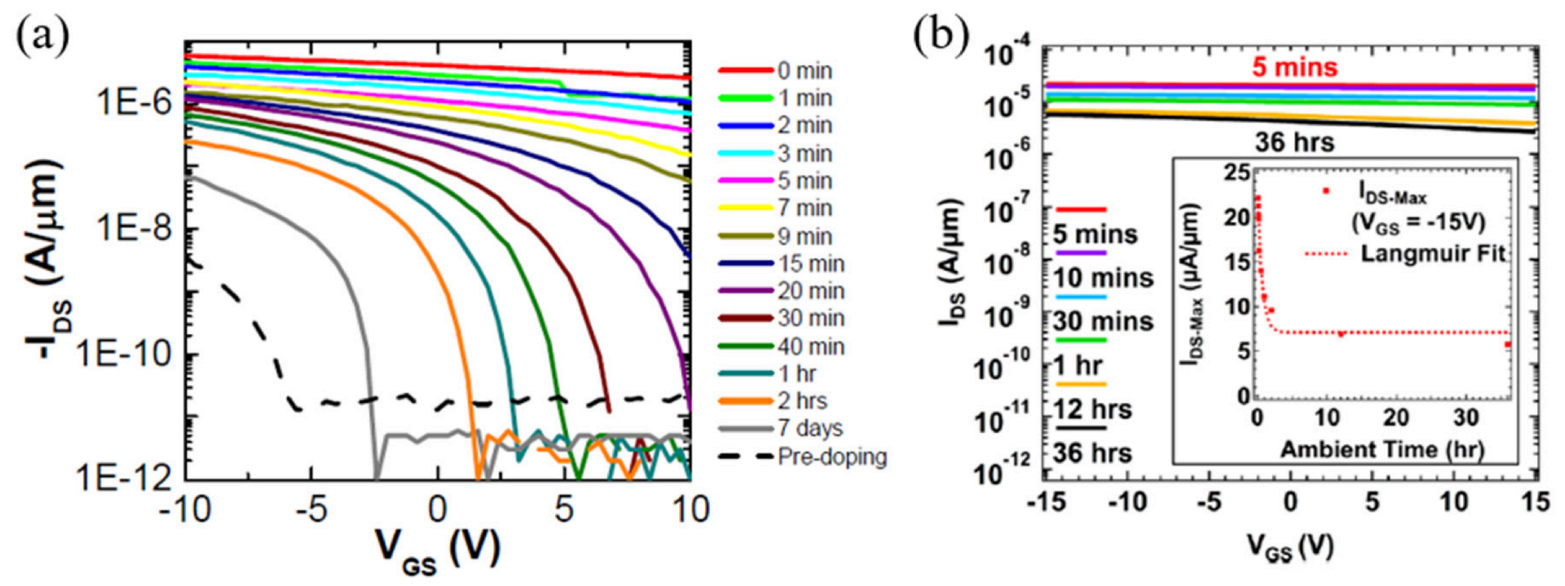
(**a**) Time-dependent transfer characteristics of monolayer WSe_2_ FETs after NO_2_ treatment and subsequent air exposure. Reproduced with permission [38]. (**b**) Transfer curves of WSe_2_ FETs treated with NO_2_ at 150 °C and measured after ambient exposure for 5 min to 36 h. The inset shows the variation in drain current with air exposure time, fitted to a time-dependent Langmuir desorption model. Reproduced with permission [24].

Although surface charge transfer by physisorbed dopants provides a sensitive and nondestructive method for modulating carrier density, it can be unstable. Because the basal plane of ideal WSe_2_ is largely free of dangling bonds, dopants may detach during exposure to ambient air, thermal stress, or lithographic solvents. Chemical bonding instead occurs preferentially at reactive defect sites, particularly V_Se_ and substituted oxygen atoms, where adsorbates can be immobilized without extensive disruption of the host lattice. This defect-selective reactivity is critical for chemical doping of WSe_2_ because it enables persistent modification of the interfacial electronic structure while largely preserving long-range crystal order. Gaseous nitrogen oxides exhibit two distinct p-doping schemes in WSe_2_. In early device studies, exposure to NO_2_ at room temperature primarily induced SCTD and was particularly useful for doping source/drain regions in the degenerate regime. However, this effect was mostly reversible because physisorbed NO_2_ gradually desorbed from WSe_2_ under ambient conditions (Figure 7a) [38]. When thermal energy is introduced between 100 and 150 °C, the interfacial chemistry changes from physisorption to strong chemisorption (Figure 7b) [24]. Under these elevated-temperature conditions, NO_2_ molecules selectively bind to native V_Se_ sites within the lattice to form stable WSe_2–x–y_O_x_N_y_ species. This self-healing process not only eliminates structural defects but also changes the electronic state. DFT calculations revealed that the NO:WSe_2_ configuration introduces distinct acceptor-like defect bands between mid-gap and E_V_. The emergence of these shallow acceptor bands pins the E_F_ deep within the valence band.

**Figure 8:**
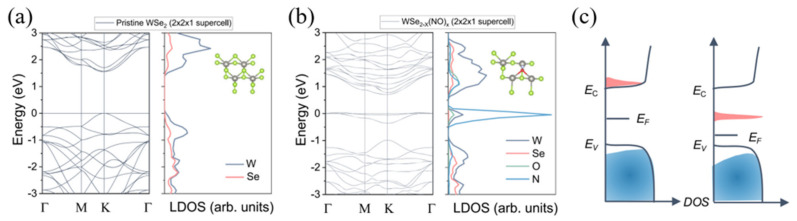
(**a**,**b**) Density functional theory (DFT)-calculated band structures (left) and local density of states (LDOS; right) for (**a**) pristine WSe_2_ and (**b**) WSe_2_ with an NO molecule occupying V_Se_ sites. The insets show the corresponding atomic configurations used in the calculations. (**c**) Schematic density of states (DOS) profiles showing the effect of NO doping. Reproduced with permission [39].

Another stable gaseous dopant scheme is elevated-temperature NO treatment. Lan et al. showed that exposing WSe_2_ to NO gas at 100 °C induces stable WSe_2–x_(NO)_x_ formation [39]. This shifts the E_F_ toward E_V_, yielding a hole concentration of 6 × 10^12^ cm^−2^ (Figure 8). In addition, V_Se_ sites can serve as chemically reactive atomic anchoring sites. Thiophenol-, ammonium sulfide-, and thiol-based functionalizations are directly associated with adsorption at V_Se_ sites in WSe_2_ [40,41,42].

#### 3.1.4. Native Oxide as a High-Quality Interfacial Layer

Unintended oxidation of semiconductor channels has traditionally been associated with interface trap states that scatter charge carriers and degrade device performance. However, research on WSe_2_ has challenged this conventional view. Controlled oxidation has emerged as an unusual but effective p-doping method because the reaction can stop after only the outermost layers are converted to tungsten oxide (WO_x_), thereby preserving the crystal structure of the underlying WSe_2_ [32].

**Figure 9:**
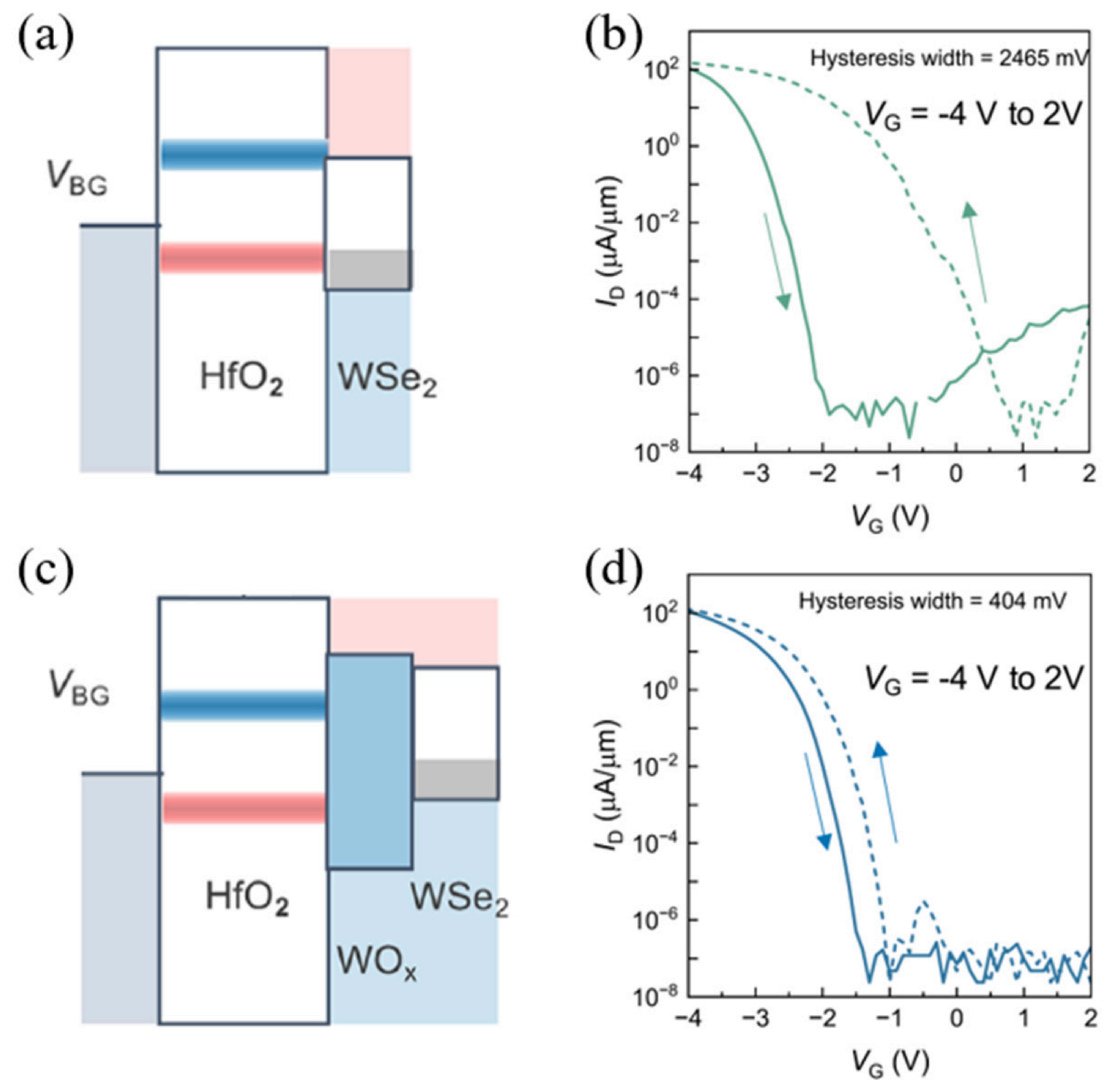
(**a**) Schematic energy-band diagram and (**b**) corresponding transfer characteristics of a WSe_2_ FET fabricated directly on an HfO_2_ gate stack. (**c**) Energy-band diagram and (**d**) transfer characteristics of a device incorporating a WO_x_ interfacial layer between WSe_2_ and HfO_2_. Reproduced with permission [43].

Recent work has extended the native-oxide strategy from doping to gate-stack design (Figure 9) [43]. By directly introducing self-limiting WO_x_ between a WSe_2_ channel and high-κ dielectrics such as HfO_2_, the native oxide performs two functions. First, it induces strong p-type charge-transfer doping and tunes V_th_. Second, it physically and electrostatically decouples the WSe_2_ channel from remote phonon scattering and defect bands associated with the HfO_2_ layer. Transistors incorporating this hybrid native-oxide interfacial layer exhibit a nearly ideal subthreshold swing (*SS*) of approximately 68 mV/dec and strongly suppressed hysteresis during gate-voltage sweeps.

### 3.2. n-Type Doping Approaches

#### 3.2.1. Alkali Metal

**Figure 10:**
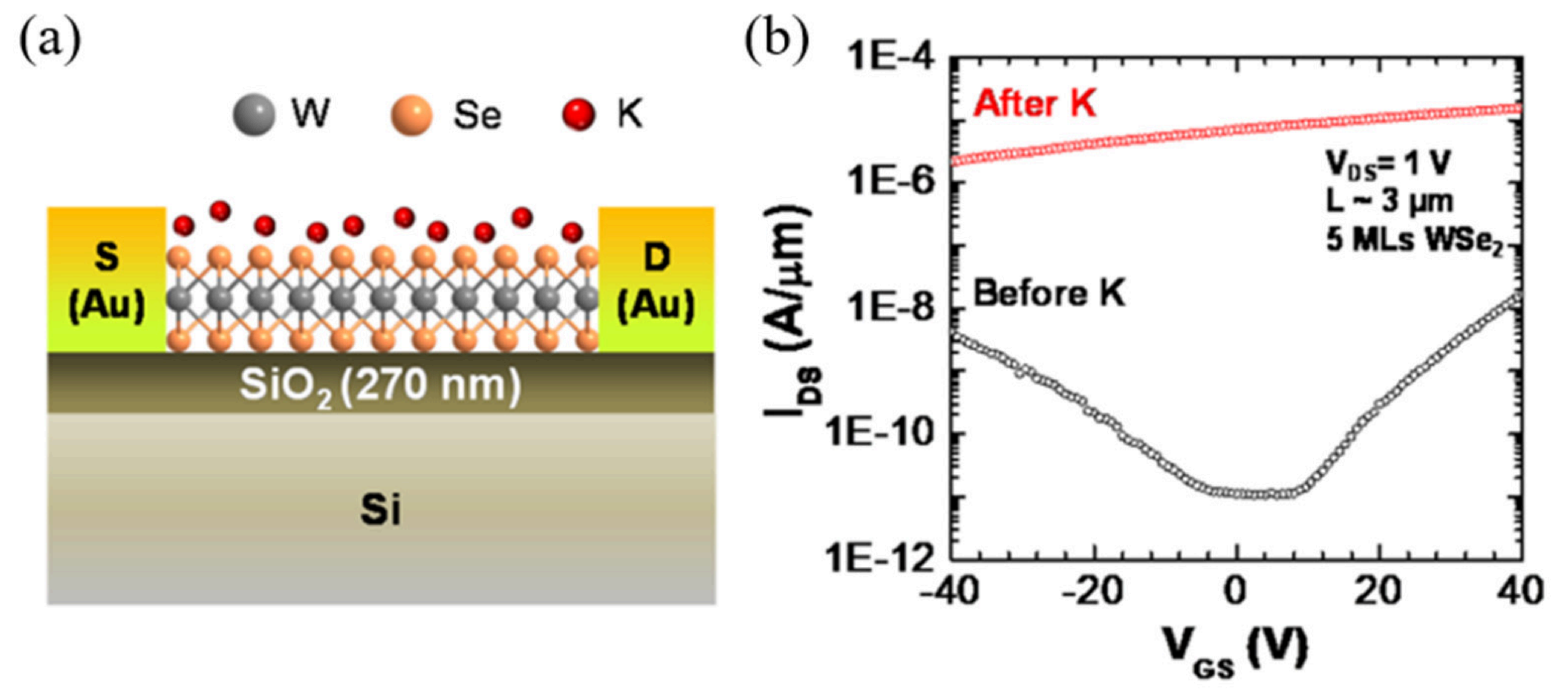
(**a**) Schematic illustration of K adsorption on the WSe_2_ surface. (**b**) Transfer characteristics measured before and after K treatment, represented by black and red symbols, respectively. Reproduced with permission [44].

Although WSe_2_ intrinsically exhibits p-type or ambipolar transport due to mid-gap E_F_ pinning against conventional metal contacts, CMOS circuits require an equally robust n-channel component. Potassium (K) has served as a typical surface donor in this context. Upon exposure to K vapor under vacuum, few-layer WSe_2_ transistors exhibit distinct n-type transport behavior with significantly increased conductance (Figure 10) [44]. Unlike physisorbed nonpolar molecules, K atoms form strong electrostatic interactions with the selenide basal plane, with a K-Se bond length of approximately 3.36 Å. DFT simulations yielded a charge transfer of roughly 0.5e from each K atom to WSe_2_. This large influx of free electrons shifts the E_F_ upward, pushing WSe_2_ into a highly degenerate n-doped regime with a sheet electron density of about 2.5 × 10^12^ cm^−2^.

#### 3.2.2. Organic Electron-Donating Molecules

Beyond highly reactive and environmentally sensitive alkali metals, organic molecules have emerged as stable and solution-processable candidates for inducing electron accumulation in TMDs. Benzyl viologen (BV) is a representative example. In its chemically reduced neutral state (BV^0^), this molecule exhibits a low reduction potential of approximately −0.79 V versus the standard hydrogen electrode (SHE) [45]. Because the E_C_ of multilayer WSe_2_ is located near 0 V versus SHE, this energy difference thermodynamically drives spontaneous two-electron transfer from a single BV molecule to the underlying WSe_2_ [31]. This interfacial electron injection effectively converts the p-type or ambipolar behavior of pristine WSe_2_ toward n-type transport. In addition, the polarity transition can be reversed by rinsing with toluene, consistent with an adsorbate-mediated process.

**Figure 11:**
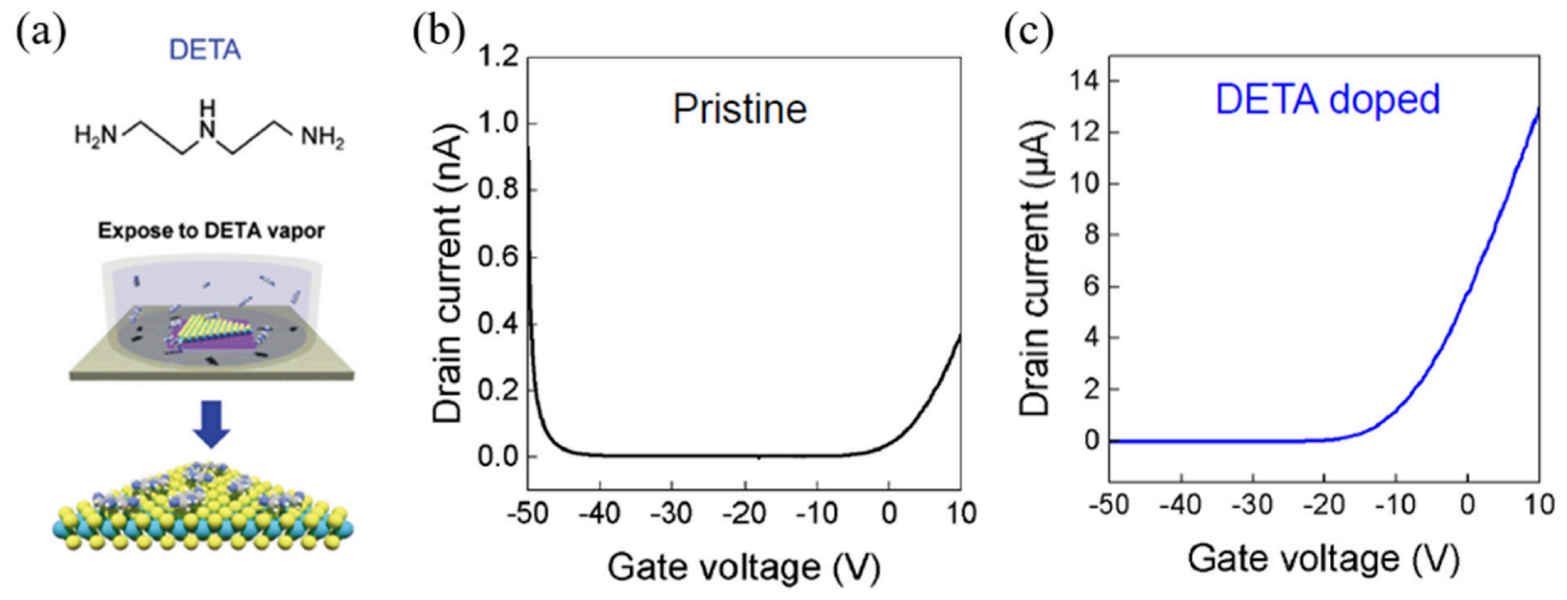
(**a**) Schematic illustration of the molecular doping process using DETA to achieve n-type WSe_2_ channels. (**b**,**c**) Transfer characteristics of a representative WSe_2_ FET measured (**b**) before and (**c**) after DETA treatment. Reproduced with permission [46].

Diethylenetriamine (DETA) provides a clearer example of n-type doping of the channel in monolayer WSe_2_ (Figure 11) [46]. Upon exposure to DETA vapor, CVD-grown WSe_2_ exhibits a negative V_th_ shift and n-type conduction with a maximum electron mobility of 25 cm^2^ V^−1^ s^−1^. X-ray photoelectron spectroscopy shows an upward movement of the WSe_2_ E_F_ after DETA treatment, whereas DFT indicates that direct electron transfer from DETA to pristine WSe_2_ is energetically unfavorable. Ji et al. therefore proposed a defect-assisted pathway in which selenium-vacancy states within the bandgap mediate charge donation to WSe_2_ [46]. Because the n-doping effect is easily removed by water rinsing, DETA is more appropriately described as a primarily physisorbed donor aided by defect states rather than as a strongly chemisorbed species.

#### 3.2.3. Lone Pair Electron Donation

**Figure 12:**
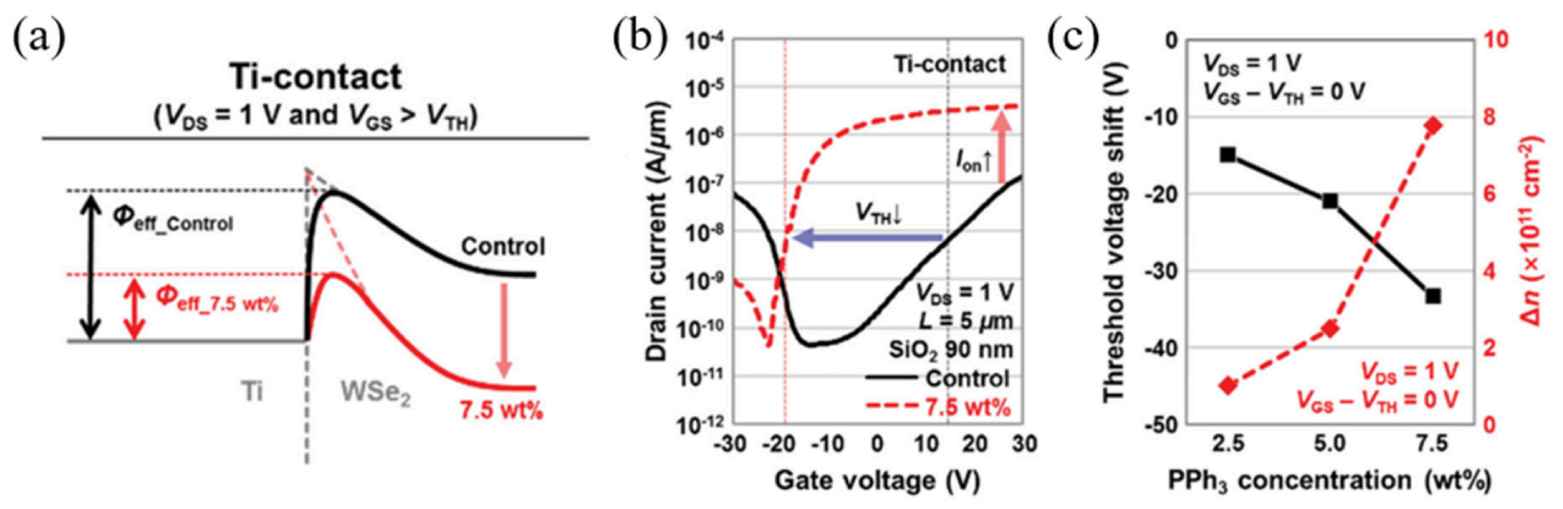
(**a**) On-state energy-band profiles of the Ti/WSe_2_ junction before (black solid line) and after (red solid line) PPh_3_ doping. (**b**) Transfer curves of the untreated control device and the transistor doped with 7.5 wt% PPh_3_, represented by the black solid and red dashed curves, respectively. (**c**) Extracted changes in V_th_ and carrier concentration for Ti-contacted WSe_2_ transistors. Reproduced with permission [47].

Beyond reactive alkali metals and conventional aliphatic amines, triphenylphosphine (PPh_3_) offers a non-destructive, controllable route for n-type modulation. The fundamental doping mechanism is related to the molecular structure of PPh_3_ [47]. Specifically, the central phosphorus atom possesses a lone pair of electrons that does not participate in covalent bonding with the surrounding phenyl rings. When PPh_3_ is spin-coated onto WSe_2_, these lone-pair electrons act as spontaneous electron donors. The electrical consequences of PPh_3_ treatment depend strongly on the contact metal. In Pt-contacted devices, increasing PPh_3_ concentration modifies E_F_, increases the effective hole-injection barrier, and decreases the hole current. In Ti-contacted WSe_2_ transistors, however, V_th_ shifts negatively, and the extracted electron density increases from 1.02 × 10^11^ to 7.77 × 10^11^ cm^−2^ (Figure 12). Compared with K doping, which can drive few-layer WSe_2_ into the metallic regime, PPh_3_ remains within the non-degenerate doping range, making it effective for controlling polarity while preserving the semiconducting nature of the channel.

#### 3.2.4. In Situ Defect Engineering

**Figure 13:**
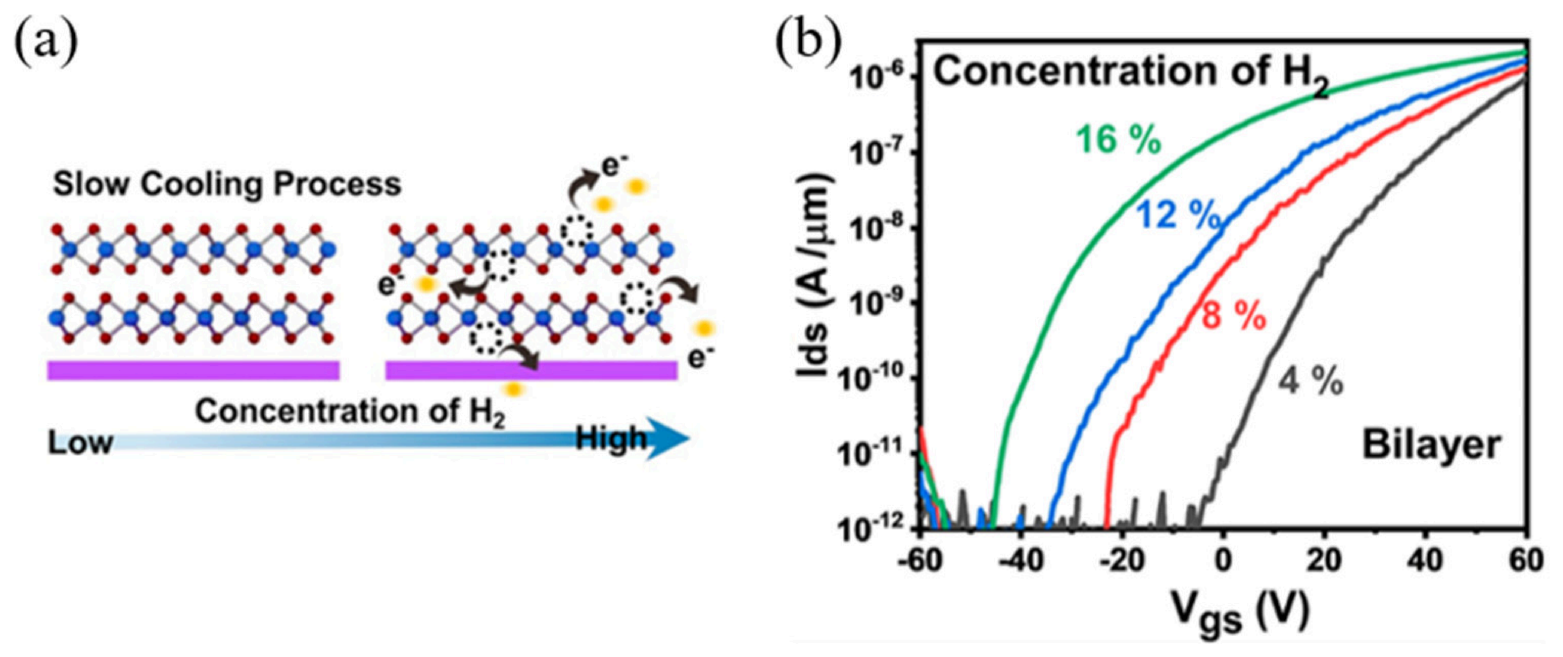
(**a**) Schematic illustration of selenium-vacancy generation during the slow-cooling stage under H_2_ gas flow. (**b**) Transfer characteristics of bilayer WSe_2_ FETs synthesized using H_2_ concentrations of 4%, 8%, 12%, and 16% during the cooling process. Reproduced with permission [34].

Post-synthesis chemical treatments effectively modulate the carrier density of WSe_2_. However, they typically require complex multi-step processing and leave surface adsorbates that degrade long-term stability. To address these extrinsic limitations, an in situ defect-engineering strategy has recently been demonstrated by modulating the H_2_ flow rate during the cooling stage of CVD growth (Figure 13) [34]. In this process, the introduced H2 induces the reduction of WSe_x_O_y_ inherently present in CVD-grown crystals. This generates selenium single and double vacancies that act as effective shallow donor states. X-ray photoelectron spectroscopy (XPS) analysis reveals a shift in the Se 3d and W 4f core levels toward higher binding energies, confirming an upward shift of the E_F_ toward the E_c_. This defect-mediated doping is highly thickness-dependent, demonstrating a complete n-type transition in bilayer WSe_2_. This is attributed to the fact that the downward shift of E_c_ in thicker films brings the conduction band closer to the vacancy-induced defect states, thereby reducing the energy barrier for free-electron injection. While increasing the H_2_ concentration from 4% to 16% increases the electron density up to 2.5 × 10^12^ cm^−2^, the field-effect mobility decreases due to the increased carrier scattering at localized defect centers.

## 4. Impact of Doping on WSe_2_ Device Physics

### 4.1. Contact Resistance (R_C_) Reduction

The fundamental bottleneck in realizing high-performance 2D WSe_2_ electronics lies in severe parasitic R_C_ localized at the metal–semiconductor junction rather than in the intrinsic channel mobility itself [48]. Owing to inevitable E_F_ pinning caused by the complex interaction between MIGS and DIGS, a strong Schottky barrier is generally formed regardless of the work function of the selected metal [49]. Such high-energy barriers severely hinder efficient charge-carrier injection, limiting charge transport in transistors and obscuring the intrinsic transport properties of atomically thin channels. High-concentration doping in the contact region is an effective strategy for lowering these interfacial barriers. When dopants induce a degenerate density of free charge carriers, such as electrons or holes, directly beneath the metal electrode, the electrostatic properties of the junction are substantially reconfigured. This high local charge density induces abrupt energy-band bending at the interface, resulting in a significant reduction in the spatial width of the depletion region [50].

In undoped WSe_2_, charge carriers must gain sufficient thermal energy to overcome a wide barrier via thermionic emission, thereby exponentially suppressing the current, especially at low operating bias. When the barrier width is narrowed to the sub-nanometer scale by degenerate doping, however, the wave-function penetration probability increases exponentially. Under high-concentration doping conditions, the transport regime can therefore transition from thermionic emission to thermionic-field emission and, ultimately, direct quantum-mechanical tunneling. This type of carrier injection can form an ohmic contact by drastically reducing R_C_. This phenomenon was experimentally verified using PtCl_4_-doped few-layer WSe_2_, for which an R_C_ of 0.23 ± 0.07 kΩ µm was obtained [37]. Doping can also reduce not only the barrier width but also the barrier height. In monolayer WSe_2_, NO chemisorption lowers the Schottky barrier height for hole injection from approximately 250 to 50 meV, reducing R_C_ to 0.88 kΩ µm. This improvement indicates that NO molecules modify interfacial pinning and barrier alignment [39]. A similar dual effect is observed in WO_x_Se_y_-based doping methods, where the WO_x_Se_y_ layer promotes tunneling current while suppressing MIGS at the interface, thereby reducing the R_C_ to 1.2 ± 0.3 kΩ µm at a hole density of 10^13^ cm^−2^ [51].

### 4.2. Carrier Mobility Enhancement

In classical semiconductor physics, the introduction of dopant atoms often degrades phonon-limited carrier mobility through ionized-impurity scattering [52,53,54]. However, in atomically thin 2D semiconductors, dopants can improve the extracted field-effect mobility because carrier transport is frequently limited not only by phonon scattering but also by carrier injection, interfacial charged impurities, and trap-assisted carrier capture [55]. Because charge carriers in WSe_2_ are confined to a sub-nanometer plane, they are strongly affected by Coulombic scattering from adjacent substrate traps, such as SiO_2_ surface charges, and intrinsic interfacial defects [17]. When a substantial concentration of free carriers is introduced into the channel by doping, these mobile charges provide electrostatic screening, reducing the long-range Coulomb potentials generated by charged impurities. Consequently, the scattering cross-section can decrease, increasing the carrier mean free path. In addition to electrostatic screening, defect-state control can also enhance field-effect mobility [56]. Pristine WSe_2_ often contains V_Se_, which acts as deep-level trap states and scatters holes. When these states are passivated or chemically healed, trap-mediated scattering is reduced, and the channel responds more uniformly to gate bias. Cho et al. showed that substitutional-oxygen-mediated healing of V_Se_ in WSe_2_ eliminates deep-level trap states and improves carrier transport [56,57]. Similarly, molecular dopants such as 4-NBD can be used to heal these defects [46]. During doping, reactive molecules preferentially anchor to vacancy sites. By removing localized trap states in the bandgap, this targeted chemisorption suppresses trap-mediated scattering and restores channel transport. As a result, this doping process can increase field-effect hole mobility by up to four orders of magnitude.

### 4.3. Degenerate vs. Non-Degenerate Doping

As noted above, degenerate doping is advantageous in the contact-access regions because a high local carrier density narrows the Schottky-barrier width and promotes carrier injection via thermionic field emission and tunneling, thereby reducing R_C_. However, when a high carrier density is introduced into the channel, gate electrostatic control is weakened. As a result, the SS degrades and the off-state leakage current increases, making the transistor inefficient for low-power switching. Non-degenerate doping is therefore more appropriate for V_th_ control within the active channel while preserving low off-state current. OTS is a representative example because it tunes the WSe_2_ hole density within the non-degenerate regime, with reported carrier densities of 2.1 × 10^11^ to 5.2 × 10^11^ cm^−2^ [58].

Accordingly, the most effective strategy for high-performance WSe_2_ transistors is spatially decoupled doping: source/drain regions are heavily doped, whereas the gated channel is kept intrinsic or lightly doped. This architecture preserves strong gate control while maintaining efficient carrier injection at the contacts. Selective oxidation and contact-spacer doping studies support this conclusion. Self-aligned p^+^-p-p^+^ WSe_2_ FETs fabricated via selective oxygen-plasma doping exhibited on/off current ratios up to 2.5 × 10^7^ with an *SS* of 98 mV/dec [37,59]. Additionally, top-gate devices doped with PtCl_4_ maintained good switching performance, exceeding an on/off current ratio of 10^7^ and an SS of 155 mV/dec at a gate length of 200 nm. More recently, a spacer-doping scheme based on self-aligned WO_x_ conversion produced a 250-fold increase in on-current while maintaining an *SS* of approximately 80 mV/dec [60]. Therefore, the practical goal for WSe_2_ is not to maximize doping concentration throughout the entire device. Instead, high carrier concentration should be localized at the contact region while maintaining effective gate control in the channel.

Table 1 presents the key performance metrics of different doping methods.

## 5. Challenges and Future Perspectives

### 5.1. Enhancing Air and Thermal Stability of Molecular Dopants

The temporal and thermal instability of doping effects by molecular dopants is mainly governed by the physisorption and chemisorption kinetics of the dopant molecules. For instance, when WSe_2_ is exposed to NO_2_ at 150 °C, the initial rapid degradation of the current occurs within 2–3 h upon subsequent exposure to ambient conditions [24]. While these physisorbed molecules are highly volatile, the molecules chemisorbed at V_Se_ sites exhibit remarkable stability, maintaining high hole current densities even after 36 h. XPS and ab initio simulations further indicate that these chemisorbed molecules form robust WSe_2–x–y_O_x_N_y_ covalent species on the surface. Owing to their high activation energy for desorption, these species enable the doping level to remain thermally stable up to 300 °C. Therefore, mitigating the desorption of weakly bound dopant molecules is crucial for maintaining long-term doping performance. Experimental studies now demonstrate several feasible stabilization routes. One practical method is to physically confine the dopant layer to prevent detachment or exposure to moisture. In WSe_2_ CMOS structures, F_4_TCNQ embedded in a PMMA matrix preserved localized p-type doping for at least two weeks in air, whereas direct molecular coating showed noticeably poorer retention [25]. The second route is defect-anchored chemisorption. High-temperature NO_x_ treatment forms stable WSe_2–x–y_O_x_N_y_ species and yields persistent p-doping; later studies using pure NO on mono- and bilayer WSe_2_ reported good retention for over 677 days [16,39,64]. A third route is oxide-assisted dopant confinement [37]. ZrO_2_ capping grown by ALD on PtCl_4_-doped WSe_2_ suppressed dopant escape and maintained the high-concentration doping state even during 300–400 °C heat treatment. Recent work also suggests that stable noncovalent doping in WSe_2_ does not require conventional redox molecules [65]. Chloroform-treated monolayer WSe_2_ exhibits long-term p-type stability exceeding eight months and thermal tolerance up to 150 °C. This exceptional stability is likely attributed to strong physisorption and potential intercalation at the WSe_2_/gate-oxide interface. This result broadens the design space for future dopants. Ultimately, future dopant engineering must focus on high electron affinity, strong adsorption, suppression of intermediate band-gap states, and compatibility with high-κ dielectric stacks.

### 5.2. Spatial Resolution: Selective Area Doping for Nanoscale Device Architectures

Uniform chemical doping across entire WSe_2_ flakes or films is unsuitable for sub-10 nm technology nodes because the gate-covered channel and ungated source/drain regions perform different electrical roles. In top-gated geometries, the extension regions must have sufficiently high carrier density to narrow the Schottky barrier and suppress R_C_ at the metal interface, whereas the channel beneath the gate must preserve strong electrostatic modulation and low off-state leakage [51,59,66]. For this reason, selective-area doping in WSe_2_ is a structural requirement rather than a general post-synthesis surface treatment. Methods for achieving such selectivity have already been demonstrated through several process routes. A self-aligned metal/h-BN gate stack can serve as an oxidation mask to create a lateral p^+^-p-p^+^ junction, in which switching is maintained by converting only oxygen-plasma-exposed spacer regions into highly p-doped regions [66]. Chen et al. developed monolayer p-FETs with sub-50 nm channel lengths by combining self-limiting oxidation of bilayer WSe_2_ with scanning-probe removal of the top WO_x_Se_y_ layer [51]. These studies demonstrated that spatially resolved doping in WSe_2_ is no longer limited to whole-channel functionalization; it can also be integrated into structures that reduce local R_C_. The remaining challenge is to place the dopant near the contact site without residue, uncontrolled diffusion under the mask, or additional damage to the monolayer. Furthermore, unintended dopant migration severely degrades device performance. Highly mobile ionic dopants presumably drift along the channel under high-field operating conditions, leading to spatial dopant redistribution and V_th_ instability. Thus, developing thermal- and field-robust doping strategies is essential for preserving high spatial resolution in nanoscale device applications. For instance, Kim et al. reported that in PtCl_4_-doped top-gated devices, the lateral diffusion of dopant ions underneath the gate dielectrics is less than 100 nm [37]. Shorter-channel WSe_2_ devices with contact-localized doping have been achieved through a combination of self-limiting oxidation and scanning probe lithography [51]. In this study, bilayer WSe_2_ was oxidized via O_2_ plasma, forming a WO_x_Se_y_ layer above an underlying monolayer WSe_2_. Subsequently, damage-free mechanical scanning probe lithography was utilized to selectively scrape away the top WO_x_ Se_y_ layer, exposing a pristine, undoped WSe_2_ channel with an ultra-short length of 50 nm. The resulting monolayer WSe_2_ FETs exhibited excellent switching behaviors with an on/off current ratio exceeding 10^7^ and a maximum drain current of 154 μA/μm.

### 5.3. Integrating Doped WSe_2_ in 3D Monolithic Architectures and Flexible Electronics

As lateral miniaturization can no longer effectively increase device density, monolithic 3D (M3D) integration is emerging as a realistic solution [67]. In this regard, WSe_2_ serves as a promising channel material because its carrier polarity and contact properties can be modulated via low-temperature chemical treatments. Recent studies showed that 2D semiconductors can be incorporated into BEOL-compatible stacks via low-temperature transfer processes that preserve underlying circuitry [68]. In particular, for WSe_2_, wafer-scale film synthesis and polarity-defined CMOS based on selective p- and n-type doping suggest that chemically tuned WSe_2_ is moving from device-level concepts toward circuit-related integration [69]. Currently, most vertical M3D demonstrations involving WSe_2_ still rely on heterogeneous stacks, typically composed of p-type WSe_2_ and n-type MoS_2_. However, in future electronic devices, M3D may be realized using p-WSe_2_ and n-WSe_2_ [70,71,72].

Flexible electronics represent another immediate application space for doped WSe_2_. Solution-processed WSe_2_ thin films have already been converted into functional p-channel transistors through molecular doping, and complementary circuits assembled with n-type MoS_2_ have delivered high inverter gain [35]. Recently, vertically stacked CMOS circuits fabricated with MoS_2_ and WSe_2_ inks have operated in a mechanically flexible form that adheres to curved surfaces through a low-temperature process at 150 °C [73]. Nevertheless, the performance limitations of such systems are still largely determined by interflake transport, defect density, R_C_, and gate-stack quality. Higher-speed all-2D architectures will depend on further progress in selective-area doping, vacancy control, van der Waals high-k dielectrics, and low-resistance vertical interconnects.

## 6. Conclusions

WSe_2_ has evolved into a practical material for polarity-tunable 2D electronic devices among representative van der Waals semiconductors. The studies discussed in this review show that controllable doping in WSe_2_ is not a secondary process step but a primary determinant of transistor operation. Because atomically thin channels are unsuitable for conventional bulk doping processes, research to date has relied primarily on surface charge transfer, defect-mediated chemical functionalization, self-limiting oxide formation, and vacancy engineering. These approaches have enabled precise control of carrier polarity, V_th_, and, most importantly, source/drain contact regions. Early air-stable WSe_2_ CMOS inverters demonstrated the feasibility of homogeneous-material complementary logic circuits, and recent wafer-scale homogeneous WSe_2_ CMOS arrays have extended this concept to inverter arrays. These results suggest that doping of WSe_2_ should be regarded not only as a means of controlling charge density but also as a practical pathway to realizing high-performance electronics based on 2D materials. Despite these advances, doped WSe_2_ should be viewed as a platform for integration rather than as a complete replacement for Si. The next stage of development depends on four key capabilities: dopants with excellent air and thermal stability; nanometer-scale selective doping; low-damage contact and dielectric integration; and wafer-scale process flows compatible with back-end thermal budgets. If these requirements are met, WSe_2_ will be highly suitable for low-power complementary logic, vertically integrated 2D/Si architectures, and flexible electronics.

## Figures and Tables

**Table 1:** Key performance metrics of various doping methods applied to WSe_2_.

Dopant	Doping Region	Sheet Carrier Density (cm^−2^)	R_C_ (kΩ μm)	SS (mV/dec)	Mobility (cm^2^ V^−1^ s^−1^)	I_on_/I_off_	Air Stability	Refs.
PPh_3_ (at RT)	Entire channel	n = 7.8 × 10^11^	-	-	18.9	10^4^	192 h	[47]
OTS (at RT)	Entire channel	p = 5.2 × 10^11^	-	-	105	10^6^	60 h	[58]
FeCl_3_ (at RT)	Entire channel	p = 5.0 × 10^12^	3.10 × 10^3^	2.6 × 10^3^	1.5	10^6^	-	[35]
F_4_TCNQ (at RT)	Entire channel	p = 3.6 × 10^12^	-	-	32.4	10^6^	-	[30]
Magic blue (at RT)	Entire channel	p = 7.0 × 10^12^	-	-	63.3	10^8^	-	[30]
Mo(tfd-COCF_3_)_3_ (at RT)	Entire channel	p = 4.2 × 10^12^	-	-	21.7	10^5^	-	[30]
4-NBD (at 60 °C)	Entire channel	p (not reported)	-	-	82	10^7^	-	[46]
DETA (at 70 °C)	Entire channel	n (not reported)	-	-	25	10^6^	-	[46]
BV (at 100 °C)	Contact region	n (not reported)	3.11	-	100	<10	100 h	[61]
OTS (at 100 °C)	Entire channel	p = 4.0 × 10^11^	-	-	168	10^7^	-	[62]
PtCl_4_ (at 130 °C)	Entire channel	p = 9.0 × 10^12^	0.32	-	-	<10	86 days	[37]
PtCl_4_ (at 130 °C)	Extension region	p = 9.0 × 10^12^	-	300	-	10^6^	-	[37]
HAuCl_3_ (at 80 °C)	Entire channel	p = 1.8 × 10^13^	0.70	-	95	<10	-	[63]
HAuCl_3_ (at 80 °C)	Extension region	p = 1.8 × 10^13^	-	89	120	10^8^	-	[63]
O_3_ (at 100 °C)	Entire channel	p = 2.6 × 10^12^	1.40	-	57	<10	10 h	[32]
NO (at 100 °C)	Entire channel	p = 1.8 × 10^13^	1.30	75	16.1	10^7^	60 days	[16]
NO (at 120 °C)	Entire channel	p = 6.0 × 10^12^	0.88	70	63	10^9^	24 days	[39]
NO_2_ (at RT)	Entire channel	p = 2.2 × 10^12^	-	-	140	<10	<10 min	[38]
NO_2_ (at RT)	Extension region	p = 2.2 × 10^12^	-	60	250	10^6^	<10 min	[38]
NO_2_ (at 150 °C)	Entire channel	p = 8.0 × 10^12^	1.27	-	-	<10	36 h	[24]
K (at RT)	Extension region	n = 2.5 × 10^12^	-	-	110	10^1^	-	[44]
H_2_ (at 870 °C)	Entire channel	n = 2.5 × 10^12^	-	-	14	10^6^	-	[34]

## Data Availability

No new data were created or analyzed in this study. Data sharing is not applicable to this article.
